# Malignancy and gene therapy in hemophilia

**DOI:** 10.1016/j.rpth.2025.103283

**Published:** 2025-12-09

**Authors:** Radoslaw Kaczmarek

**Affiliations:** Department of Pediatrics, Herman B Wells Center for Pediatric Research, Indiana University School of Medicine, Indianapolis, Indiana, USA

**Keywords:** AAV, AAV oncogenicity, AAV tumorigenicity, gene therapy, hemophilia, vector integration

## Abstract

Despite the misconception that adeno-associated virus (AAV) gene therapy vectors are nonintegrating, they can integrate into the host genome at a low but nonnegligible frequency, posing a theoretical risk of tumorigenesis. While AAV integration can trigger hepatocellular carcinoma in mice, no such association has been established in humans. None of the 10 cancer cases reported in AAV vector recipients so far has shown evidence that AAV integration drives tumorigenesis. However, the strength of the evidence from molecular analyses differed significantly across these cases. The scope and conclusiveness of causality assessments depended on sample quality and cross-validation using complementary analytical methods. For example, poor sample quality precluded a conclusive analysis in a case of a spinal cord tumor. Conversely, comprehensive analyses provided strong evidence that AAV integration was not the causative factor in a case of hepatocellular carcinoma. These findings underscore the need for standardization, global long-term follow-up, and careful communication of outcomes.

The 4 approved adeno-associated viral (AAV) gene therapies for hemophilia (3 in the US and Europe, and 1 in China) use recombinant AAV vectors to deliver therapeutic transgenes to hepatocytes [[1], [2], [3], [4]]. Despite limitations, such as variability and unpredictability of efficacy, these therapies meaningfully improve outcomes in a significant number of vector recipients, with one-third of individuals achieving normalized hemostasis with etranacogene dezaparvovec [5]. However, uncertainties regarding efficacy and safety necessitate a careful benefit-risk assessment and informed decision-making by people with hemophilia and their healthcare providers [6]. AAV vectors are typically described as nonintegrating since they form episomes to establish long-term transgene expression, unlike lenti- and retroviral vectors, which integrate into the host genome. Nonetheless, AAV vectors also integrate at a frequency estimated to be between 0.5 and 16 integration sites per 1000 cells [7]. This seemingly low rate of integration translates into potentially millions of integration events per person receiving clinical vector doses. Molecular analyses have not found evidence that vector integration drives tumorigenesis in any of the 8 cancer cases in clinical gene therapy for hemophilia or in 2 cases of spinal muscular atrophy, although the methods and certainty of conclusions differed markedly across those studies.

Several observations merit vigilance and behoove the field to heed the theoretical risk of gene therapy-driven malignancy. Multiple studies have seen hepatocellular carcinoma (HCC) in mice that received recombinant AAV, with an incidence as high as 50% to 70% compared with a background incidence below 10% [8]. These studies had several things in common. First, integrations were found in the RNA imprinted and accumulated in the nucleus (*Rian*) locus. Rian is a long noncoding RNA that plays a role in epithelial-to-mesenchymal transition, negative regulation of the Notch signaling pathway, and negative regulation of stellate cell activation. Mutations in Rian have been found to cause cancer in mice in contexts other than AAV transduction. Humans have an ortholog of Rian, called *MEG8*, but microRNA genes within that locus differ markedly from those in mice, which might explain why this locus is not a hot spot for integration in humans. This is important because upregulation of *MEG8* has correlated with poor prognosis in people with HCC [9]. Another common aspect in the mouse studies was that cancers occurred primarily in mice that received AAV as neonates. Adult animals developed HCC only if there was concomitant liver injury [10]. No other animal models, including rats, cats, dogs, and nonhuman primates, have shown evidence of AAV-triggered malignancy [[11], [12], [13], [14]]. Nevertheless, evidence from mouse models demonstrates that AAV integration is mechanistically capable of triggering malignancy. Another observation that calls for caution is insertional oncogenesis seen with retroviral vectors in humans. Notably, recombinant AAV integration is a largely nonspecific secondary event resulting from the erroneous repair of pre-existing DNA damage, in which the host's DNA repair machinery incorporates AAV DNA. This contrasts with retroviral vectors, which encode their own enzymes that catalyze integration. Despite the distinct mechanism and the absence of clear oncogenic signaling in nonmurine animal models and humans, the theoretical risk of tumorigenesis, given the sheer number of integration events, mandates rigorous malignancy surveillance in clinical trials and postapproval follow-up.

Of the 10 cancer cases in clinical AAV gene therapy [[15], [16], [17], [18], [19], [20], [21], [22]], only 1 was HCC, in contrast to mouse studies, which predominantly developed it. Despite their liver tropism, AAV vectors delivered intravenously reach most other tissues, where they may enter cells and integrate into the genome. Consequently, malignancy surveillance has not been limited to the liver. Molecular analyses of clinical cancer cases in AAV gene therapy recipients used different approaches to analyze vector integrations, and although all studies concluded that there was likely no relationship between tumorigenesis and vector integration, they did not reach these conclusions with the same level of confidence (Table).

**Table:** Malignancies reported in clinical adeno-associated viral gene therapy.

Gene therapy	Indication	Age at diagnosis	Time after dosing	Tumor	Vector integration analysis	Strength of evidence supporting the conclusions
BAX335 (AAV8-FIX-Padua)	HB	69 y	NR	Squamous cell tonsillar carcinoma	LAM-PCR	Moderate
Valoctocogene roxaparvovec (AAV5-FVIII)	HA	47 y	5 y	Acinic cell carcinoma	TES	Moderate
		20 y	3 y	B-cell acute lymphoblastic leukemia	ddPCR, WGS	Strong
UCL/St. Jude (AAV8-FIX)	HB	74 y	11.6 y	Prostate carcinoma	ddPCR	Weak
		44 y	7 y	Nonmucinous lung adenocarcinoma	ddPCR	Weak
Onasemnogene abeparvovec (AAV9-SMN)	SMA	16 mo	1.2 y	Spinal cord epithelioid neoplasm	LM-PCR	Weak
		2 y	8 mo	Pilocytic astrocytoma	ddPCR, LM-PCR	Weak
Etranacogene dezaparvovec (AAV5-FIX-Padua)	HB	69 y	1 y	HCC	LAM-PCR, TES, WGS, RNA sequencing, transcriptome profiling	Very strong
		NR	4 y	Schwannoma	NR	Unknown
		NR	4 y	Myelodysplastic syndrome	NR	Unknown

ddPCR, digital droplet polymerase chain reaction; HA, hemophilia A; HB, hemophilia B; HCC, hepatocellular carcinoma; LAM-PCR, linear amplification-mediated polymerase chain reaction; LM-PCR, ligation-mediated polymerase chain reaction; NR, not reported; SMA, spinal muscular atrophy; TES, target-enrichment sequencing; WGS, whole-genome sequencing.

Two participants of the clinical program evaluating valoctocogene roxaparvovec for hemophilia A developed cancer after AAV vector dosing. Salivary gland cancer was diagnosed in a participant in the phase 1/2 study 5 years after he received his vector dose, 6e13 vg/kg [16]. He had a history of hepatitis C, but no known acinic cell carcinoma risk factors. The tumor was removed and analyzed using target-enrichment sequencing, which found multiple vector integrations but no evidence of clonal expansion. There were no insertions within 100 kb of acinic cell carcinoma driver genes, and insertions found within 100 kb of any cancer-associated genes were most likely present in the healthy surrounding tissue, not in the tumor cells. There was 1 insertion in a tumor sample near the salivary gland tumor-associated gene (KIT), but it was far downstream of the gene and therefore unlikely to have upregulated it. Notably, the integration frequency was about 30-fold lower than that observed in the liver, consistent with AAV liver tropism. Low tumor purity (carcinoma cells accounted for only 2% of the total cells in the samples) precluded a more comprehensive analysis, which might otherwise have identified somatic driver mutations. The other case in the valoctocogene roxaparvovec program was a B-cell acute lymphoblastic leukemia diagnosed in a young participant in the pivotal phase 3 study. Here, a better-quality sample (90% tumor cells) permitted whole-genome sequencing, which identified somatic driver mutations in *JAK2* and *IZKF1* [17].

One of the 2 children diagnosed with cancer following gene therapy for spinal muscular atrophy using onasemnogene abeparvovec developed a spinal cord epithelioid neoplasm. The tumor sample quality was insufficient for conclusive molecular analysis; too little DNA was extracted from the tumor samples (10-fold below the minimum required for reliable analysis), and the average read length in ligation-mediated polymerase chain reaction was too short to efficiently detect vector-genome junctions with the primers designed for this analysis. Consequently, the analysis was unable to produce meaningful integration numbers and likely underestimated them. Additionally, the limited sample quality precluded validation with other assays [18].

The single case of HCC in the clinical program evaluating etranacogene dezaparvovec was diagnosed 1 year after vector dosing in a 69-year-old patient with a history of hepatitis B and C and metabolic dysfunction-associated steatotic liver disease (MASLD). The primary tumor could not be resected due to its location, but a secondary tumor was identified and removed for molecular analysis. Since the patient then underwent chemoembolization and a liver transplant, the primary tumor was no longer fit for analysis, but the secondary tumor produced high-quality samples that permitted a comprehensive analysis using multiple different assays, which examined vector copy numbers of episomal and integrated forms, integration sites, somatic driver mutations, and gene expression levels. Integrations were found in both tumor and noncancerous tissues at genomic sites not previously associated with HCC, and no signs of clonal expansion were detected. There was a deletion on chromosome 8 and mutations in 3 genes (*TP53*, *NFE2L2,* and *PTPRK*) that are common in HCC. Additionally, several genes (*COL1A1*, *LCN2*, *AEBP1,* and *CRP*) known to be differentially expressed in HCC were upregulated. Altogether, these results supported the conclusion that AAV integration was unlikely to have caused HCC [15].

The challenges and outcomes of these molecular studies highlight several important considerations. The methods used in vector integration analysis vary in their sample quality requirements; whether tumor samples meet those requirements determines the scope of analysis and how meaningful and conclusive it is in terms of causality assessment. Individual methods provide limited data, and ideally, a molecular analysis should involve several different methods that provide complementary information. Efficient communication among reporting physicians, pathologists, and manufacturers is key to ensuring optimal timing of tissue collection (when feasible without compromising cancer treatment or patient care), as well as optimal tissue quantity and purity, sample format, and availability of healthy comparison tissue. The approved products list contact information specifically for reporting malignancies and provides instructions for collecting patient samples for integration analysis.

These cases also highlight the importance of contextual assessment of individual cases in relation to the increased baseline risk of HCC in the general hemophilia population (due to a history of hepatitis B and C infections) and the prevalence of other risk factors in the general population (eg, MASLD). Contextualizing the baseline malignancy risk requires accounting for the declining yet persistent sequelae of past hepatitis C and B infections and the high prevalence of MASLD in people with hemophilia. Both comorbidities independently predispose this cohort to HCC, confounding long-term safety monitoring of liver-directed gene therapies [7]. Therefore, a comprehensive assessment should review the available literature and registries and consider demographics and medical history. Long-term follow-up is critical for malignancy surveillance since tumorigenesis may occur years or decades after vector infusion. Other potential mechanisms of AAV gene therapy-driven tumorigenesis, such as HCC in the context of ectopic expression and misfolding of factor VIII in hepatocytes, recently described in mice, further underscore the need for malignancy surveillance and long-term follow-up in clinical AAV gene therapy [23]. Hemophilia, as a rare disease, requires a global approach to data collection to permit robust analysis of rare events. To that end, the World Federation of Hemophilia established the global Gene Therapy Registry [24,25]. The U.S. Food and Drug Administration miscategorized AAV as nonintegrating vectors, for which it requires up to 5 years of follow-up, whereas it requires 15 years for integrating vectors, and allows a risk-based approach for vectors capable of latency or long-term expression without integration [26]. The European Medicines Agency requires up to 15 years of follow-up for medicinal products that use integrating vectors or have the potential for latency followed by reactivation, without specifying the duration of follow-up for AAV vectors. The European Medicines Agency acknowledges the need to follow patients beyond marketing authorization to answer questions about long-term safety and efficacy [27].

Furthermore, the discrepancy in methodological rigor between molecular analyses of malignancies reported so far shows a need to better define communication goals when cancer occurs. All the reports concluded that cancer was unlikely to be related to gene therapy, but this meant something different in each case. It meant that no integrations were found, that integrations were found but did not appear to have triggered tumorigenesis, or that no conclusive evidence was found because molecular analysis failed to provide meaningful data, among several other scenarios. For the sake of transparency, reporting teams must find a way to turn the complex outcomes of molecular analyses into accessible information without ignoring important nuances and sending messages that are too simple to be helpful.

The need for better communication will remain especially important as long as the field of vector integration analysis is fraught with challenges related to the lack of standardization [28,29]. The International Society on Thrombosis and Haemostasis Scientific and the Standardization Committee Working Group on Gene Therapy have spearheaded multiple standardization initiatives. Consensus protocols are needed in several areas, including tissue sample collection and processing, assay methodology selection, bioinformatics analysis, and data reporting [28]. As the field evolves, efforts toward standardization should continue, because AAV vectors, despite commercial setbacks, remain essential tools in current and many emerging gene therapies, including gene editing, which is designed to integrate transgenes into the genome. Gene editing technologies may produce off-target effects, which raises additional questions about malignancy risk. Thus, analytical standards developed now will likely remain relevant for years to come and will be foundational for addressing future technological challenges [30].
